# Mass gathering enhanced syndromic surveillance for the 8th Micronesian Games in 2014, Pohnpei State, Federated States of Micronesia

**DOI:** 10.5365/wpsar.2016.7.4.001

**Published:** 2018-03-21

**Authors:** Paul White, Salanieta Saketa, Eliaser Johnson, Sameer V. Gopalani, Eliashib Edward, Charles Loney, Alize Mercier, Tebuka Toatu, Richard Wojcik, Sheri Lewis, Damian Hoy

**Affiliations:** a ELC Programme, PHEP Office, Commonwealth Health Care Corporation, Saipan, Commonwealth of the Northern Mariana Islands.; b Research Evidence and Information Programme, Public Health Division, the Pacific Community.; c Division of Primary Health Care, Pohnpei State, Federated States of Micronesia.; d Department of Health and Social Affairs, Government of the Federated States of Micronesia.; e Medical Records Unit, Pohnpei State Hospital, Federated States of Micronesia.; f Applied Physics Laboratory, Johns Hopkins University.

## Abstract

Pohnpei State’s Division of Primary Health Care implemented enhanced surveillance for early warning and detection of disease to support the 8th Micronesian Games (the Games) in July 2014.

The surveillance comprised 11 point-of-care sentinel sites around Pohnpei, Federated States of Micronesia, collecting data daily for eight syndromes using standard case definitions. Each sentinel site reported total acute care encounters, total syndrome cases and the total for each syndrome. A public health response, including epidemiological investigation and laboratory testing, followed when syndrome counts reached predetermined threshold levels.

The surveillance was implemented using the web-based Suite for Automated Global Electronic bioSurveillance Open-ESSENCE (SAGES-OE) application that was customized for the Games. Data were summarized in daily situation reports (SitReps) issued to key stakeholders and posted on PacNet, a Pacific public health e-mail network.

Influenza-like illness (ILI) was the most common syndrome reported (55%, *n* = 225). Most syndrome cases (75%) were among people from Pohnpei. Only 30 cases out of a total of 408 syndrome cases (7%) presented with acute fever and rash, despite the large and ongoing measles outbreak at the time. No new infectious disease outbreak was recorded during the Games. Peaks in diarrhoeal and ILI cases were followed up and did not result in widespread transmission.

The technology was a key feature of the enhanced surveillance. The introduction of the web-based tool greatly improved the timeliness of data entry, analysis and SitRep dissemination, providing assurance to the Games organizers that communicable diseases would not adversely impact the Games.

## Introduction

The 8th Micronesian Games took place in Pohnpei, Federated States of Micronesia, from 19 to 29 July 2014. Participants came from the six Micronesian island countries and territories: Guam, Kiribati, the Marshall Islands, the Federated States of Micronesia, Nauru, the Commonwealth of the Northern Mariana Islands and Palau.

Pohnpei is a small island state of 35 981 people (2010 census). The 8th Micronesian Games held in the area around Kolonia, the main town of Pohnpei, attracted approximately 1700 athletes and officials (Saketa S, Public Health Division, Pacific Community, unpublished report, 2014). This population influx posed public health risks for the introduction of communicable diseases, (*1*) as illustrated by a widespread measles outbreak in Pohnpei two months before the Games. This had the potential to overwhelm health services, disrupt the Games and trigger the spread of measles across the Federated States of Micronesia and the wider region.

To mitigate disease risks and aid identification of adverse health events, an enhanced syndromic surveillance system for mass gatherings was implemented by Pohnpei’s Division of Primary Health Care (DPHC) in partnership with the country’s Department of Health and Social Affairs (DHSA), the Pacific Community (SPC) and Johns Hopkins University (JHU). Enhanced surveillance for mass gatherings is increasingly used in large developed nations. (*2*) In the Pacific, mass gathering surveillance was used for the 2012 11th Festival of Pacific Arts, Solomon Islands and the 2013 Pacific Mini Games, Wallis and Futuna. (*3*, *4*) Here we describe the enhanced surveillance system implemented by Pohnpei State and discuss some of the sustainable benefits arising from the mass gathering surveillance experience.

## Methods

SPC has developed a three-stage strategy for preparing for and implementing enhanced surveillance for mass gatherings. This approach is summarized in a process map (available online). (*4*) The preparation stage includes a disease risk assessment and an assessment of the existing surveillance system to meet the mass gathering surveillance needs. Pohnpei implemented an early warning syndromic surveillance system in 2010 as part of the World Health Organization (WHO)/Pacific Public Health Surveillance Network Pacific Syndromic Surveillance System. (*5*) Data are collected daily from the central public hospital (Pohnpei State Hospital) and weekly from the private hospital (Genesis Hospital) for acute fever and rash, prolonged fever, influenza-like illness and diarrhoea syndromes covering important outbreak-prone diseases in Pohnpei State. A weekly surveillance report is disseminated to DHSA and WHO. As an early warning system, the syndromic surveillance system allows the Federated States of Micronesia to meet indicator-based surveillance requirements under the International Health Regulations (2005). (*6*) This system was the foundation for the mass gathering surveillance implemented for the Games.

For the Games, the number of sentinel sites was expanded to 11, comprising Games venues, hospitals and community clinics distributed around Pohnpei, the number of syndromes was increased to eight (diseases recorded in parentheses):

- acute fever and rash (AFR) (measles, dengue, rubella, meningitis, leptospirosis);
- influenza-like illness (ILI) (influenza and other viral or bacterial respiratory diseases);
- prolonged fever (typhoid fever, dengue, leptospirosis, malaria);
- fever and jaundice (hepatitis A infection);
- watery diarrhoea (cholera);
- non-watery diarrhoea (viral or bacterial gastroenteritis, including food poisoning and ciguatera fish poisoning);
- foodborne disease outbreak (salmonella, *Staphylococcus*, *Clostridium*, *Campylobacter* and rotavirus infections); and
- heat-related illness (heat cramps, heat exhaustion and heat stroke).

All sentinel sites except the two Games venues were community medical providers operating within their normal hours. Of the two Games sites, the Pohnpei Island Central School (PICS) High School provided a temporary clinic at the Games village for primary care services to athletes and officials, operating seven days a week. The second Games site, the College of Federated States of Micronesia-FSM dispensary, provided acute, preventive health care and counselling staffed by a full-time nurse and was open Monday to Friday during normal working hours. All sentinel site staff underwent a two-day surveillance training that focused on understanding the syndrome case definitions, accurate completion of the surveillance register and laboratory specimen collection and referral processes.

Each sentinel site manually completed a daily surveillance form that recorded the number of acute care encounters and syndrome cases; the completed forms were collected daily from each sentinel site. The data were entered into the Suite for Automated Global Electronic bioSurveillance OpenESSENCE (SAGES-OE) open-source, web-based application. Developed by JHU, SAGES-OE was designed for surveillance and epidemiological analysis particularly in resource-constrained settings. (*7*) It was adapted for the Micronesian Games by JHU in partnership with SPC. Drop-down lists for key variables facilitated efficient data entry and helped to ensure the completeness and consistency of data. When incomplete data were discovered, the relevant sentinel site was contacted. It took approximately five hours each day to collect and enter data.

SAGES-OE analysis and visualization tools were used to summarize the epidemiological situation that was reported in daily situation reports (SitReps). These included totals for encounters and syndromes together with epidemic curves used to track syndrome trends. SitReps were distributed to stakeholders in the DHSA and the Games organizing committee, and were posted to the Pacific health e-mail network, PacNet. The surveillance was operational from 17 July – two days before the Games – until 6 August, one week after the Games. Eighteen daily SitReps were produced.

### Ethics

Ethics committee approval was not required.

## Results

**Fig. 1:**
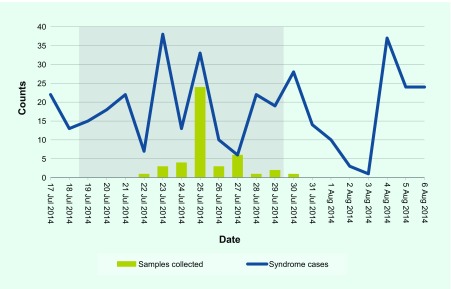
Comparison of daily syndrome counts and laboratory sample submissions, Pohnpei State, 17 July to 6 August 2014 (dates of Games shaded)

There were 5640 encounter cases and 408 syndrome cases from the 11 sentinel sites during the 21 day surveillance period. Sentinel site encounters ranged from 52 to 2040 with a median of 165 and mean of 496. The variance in encounters reflects the highly variable catchments of hospital outpatient departments and smaller community clinics. The Games-specific sentinel site (PICS High School) recorded 3% of all encounters (*n* = 165) and 3% of all syndromic cases (*n* = 13). One site (Wone dispensary) reported no syndromes. **Table 1** summarizes acute care encounters and syndrome presentations by sentinel site from 17 July to 6 August 2014. There was a high degree of variation in daily syndrome presentations (**Fig. 1**). Overall, syndrome cases represented 7% of encounters (ranging from 2% to 18%). Residents of Pohnpei reported more syndromes (75% of all syndromes, *n* = 305) than those from outside Pohnpei.

**Table 1:** Summary total encounters and syndromes by reporting sentinel site, Pohnpei State, 17 July to 6 August 2014

Sentinel site	Number encounter cases*	Number syndrome cases^#^ (% of encounters)	Syndrome cases per 1 000 encounters	Acute fever and rash	Watery diarrhoea	Non-watery diarrhoea	Influenza-like illness	Prolonged fever	Fever and jaundice	Heat-related illness	Foodborne disease syndrome
Berysin CHC	66	3 (5%)	45.5	0	1	0	2	**0**	**0**	**0**	**0**
Genesis Hospital	1139	31 (3%)	27.2	2	10	1	18	**0**	**0**	**0**	**0**
Medpharm CHC	145	7 (5)	48.3	0	0	4	3	**0**	**0**	**0**	**0**
PICS High School	165	13 (8%)	78.8	0	5	0	5	**2**	**0**	**1**	**0**
Pohnpei CHC	1157	202 (18%)	174.6	1	24	11	159	**6**	**0**	**1**	**0**
Pohnpei State Hospital	2040	126 (7%)	61.8	26	49	9	35	**6**	**1**	**0**	**0**
Sokehs Dispensary	52	4 (8%)	76.9	1	1	2	0	**0**	**0**	**0**	**0**
COM Dispensary	63	4 (6%)	63.5	0	2	2	0	**0**	**0**	**0**	**0**
Lukop Dispensary	95	8 (8%)	84.2	0	4	2	2	**0**	**0**	**0**	**0**
Pohnlangas Dispensary	512	10 (2%)	19.5	1	3	5	1	**0**	**0**	**0**	**0**
Wone Dispensary	206	0 (0%)	0	0	0	0	0	**0**	**0**	**0**	**0**
**TOTAL**	**5640**	**408 (7%)**		**31**	**99**	**36**	**225**	**2**	**1**	**1**	**0**

* Encounter cases represent all acute care cases visiting a clinic regardless of whether they have one or more of the eight syndromes; e.g. a sprained ankle would be included in the encounter case count.

^#^ Syndrome cases represent all acute care cases visiting a clinic who have one or more of the eight syndromes; e.g. a sprained ankle would NOT be included in the syndrome case count.

CHC = Community Health Center

COM = College of Federated States of Micronesia

The three larger Kolonia town sentinel sites, Pohnpei State Hospital, Genesis Hospital and Pohnpei/Kolonia Community Health Center, accounted for over three quarters of all encounters (77%, *n* = 4336) and a larger proportion (88%, *n* = 359) of all syndrome cases. Pohnpei State Hospital had a third of encounters (36%, *n* = 2040, 61.8 per 1000 encounters) and syndromes (31%, *n* = 126). Pohnpei Community Health Center had the second greatest number of encounters (21%, *n* = 1157), 15% lower than the State Hospital, but it had a greater catchment of cases accounting for half of all syndromes (50%, *n* = 202) and by far the highest rate of syndromes (174.6 per 1000 encounters). The private Genesis Hospital accounted for the third greatest number of encounters (20%, *n* = 1139), but it had a far lower surveillance sensitivity with 8% (*n* = 31) of syndrome cases (27.2 per 1000 encounters).

ILI was the most prominent syndrome accounting for half of all cases (55%, *n* = 225) followed by watery diarrhoea (24%, *n* = 99), non-watery diarrhoea (9%, *n* = 36) and AFR (8%, *n* = 31). There were two prolonged fever cases. Both fever and jaundice and heat-related illness had one case. No foodborne disease outbreak was reported. Watery diarrhoea (24%) and AFR (8%) accounted for almost one third (32%) of all reported syndromes and are indicative of more severe diseases requiring urgent attention. These cases were prioritized and followed up by the response team.

A marked difference in the sex distribution of syndrome cases was noted in the four sentinel sites in the rural areas with nearly three quarters of cases being female (72%). Whereas the seven sentinel sites in the more populated or urban areas (see **Table 1**) had roughly an equal male (52%) and female (48%) representation.

**Fig. 2:**
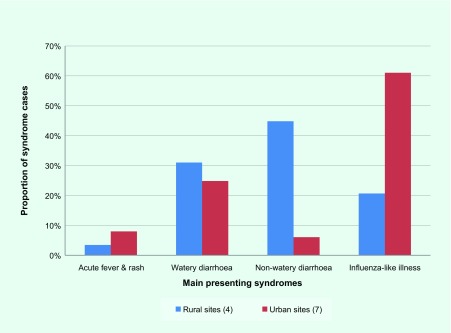
Distribution of syndrome cases reported in rural and urban point-of-care sentinel sites, Pohnpei State, 17 July to 6 August 2014

The urban–rural difference was also evident in the distribution of syndromes. **Fig. 2** describes the distribution of the four main syndromes, AFR, watery and non-watery diarrhoea, and ILI, in the seven urban area sentinel sites and in the remaining four rural sites. In the urban areas, ILI was the largest syndrome burden (61% of urban syndromes); in the rural areas, non-watery and watery diarrhoea were the two main presenting syndromes (75% of rural syndromes), while ILI was the third highest represented syndrome (21%).

Between 22 and 30 July, 45 clinical samples were collected, comprising 23 nasopharyngeal swabs/aspirate samples for ILI testing and 22 stool samples for watery and non-watery diarrhoea testing. **Fig. 1** illustrates the large sample collection peak on 25 July, while the remaining days produced fewer samples.

## Discussion

The enhanced surveillance implemented at the 8th Micronesian Games provided important data for public health security reassurance for the Games organizers. The SitReps were well received by the Pacific public health community who posted positive comments on PacNet (personal communication with Dr Eliaser Johnson, DPHC, Pohnpei State). The 31 cases of AFR were followed up, and the ongoing measles outbreak had no detrimental impact on the Games. The sentinel sites demonstrated good surveillance coverage and sensitivity; the Games-specific site had the third highest rate of syndrome cases (78.8 per 1000 encounters) but with only 3% (2.9%) of all encounters. This was greater than Pohnpei State Hospital, which had the largest number of encounters (36%) but fewer syndrome cases (61.8 syndrome cases per 1000 encounters).

The web-based SAGES-OE system enabled easy data entry, data storing, data collation and analysis and accelerated SitRep production as multiple users could access data simultaneously. The cloud storage feature helped to reduce local storage server costs as well as increase efficiency and off-site security. However, while SAGES-OE was used in the Pohnpei public health office and regional SPC office, it could not be implemented as a fully networked sentinel site data entry system due to a lack of computers and computer-trained staff. More challenging was the lack of connectivity to all health-care facilities, especially in the rural areas where wireless connectivity is hampered by low or no coverage. Nevertheless, as a public health tool, SAGES-OE is one of the key success factors of the surveillance and features in the post-Games surveillance sustainability plan of DPHC, where the goal is achieving an integrated surveillance system that allows daily data entry at sentinel sites.

Beyond outbreak detection there are additional benefits to population-representative surveillance data. Surveillance data have intrinsic value as indicators of health service performance. (*8*) The regular collection of syndromic surveillance data serves as a powerful evidence base that can be exploited for better-informed health planning and decision-making. (*9*) This includes understanding demands on laboratory services during peak times and understanding differences in disease burdens across the population.

The laboratory sample submissions data identified the unequal distribution of samples sent to the laboratory compared to daily syndrome activity (**Fig. 1**). The single peak in clinical sample collection contrasts with the greater variation of daily syndrome counts. Identifying the mismatched laboratory sample collection to syndrome case activity can be used to demonstrate to sentinel sites the importance of more regular sample collection as an effective tool to support public health surveillance (i.e. matching sample collection to syndrome activity).

The urban–rural syndrome differences can help identify important public health gaps for better prioritized and more efficient interventions and use of resources. For example, diarrhoea from polluted water sources might be more prevalent in rural areas. Whereas a greater ILI burden in urban areas indicates greater respiratory disease transmission in more densely populated areas while also indicating clean water access and waste water treatment that reduce diarrhoea episodes. This assists prioritization as watery diarrhoea can be indicative of severe diseases requiring urgent attention, particularly in children, indicating the need for different health promotion messaging and interventions. Finally, this information assists in identifying the appropriate type of public health interventions, for example joint public and environmental health responses to watery diarrhoea outbreaks, whereas a joint response is not necessary for ILI.

### Challenges and lessons learnt

The enhanced surveillance system implemented for the 8th Micronesian Games demonstrated the need for good planning and preparation including a substantial lead time of at least 12 months to establish and test the web-based surveillance tools; and for areas with low connectivity, to test methods for timely manual data collection. The enhanced surveillance also indicated the importance of adequate staff resourcing to address staff fatigue caused by the intense daily operation of the surveillance for multiple weeks. This point demonstrates the value of a joint implementation in small-island states where resources are limited. The joint implementation with SPC leveraged capability (expertise) and capacity (extra people). Based on the existing syndromic surveillance, the Pohnpei DPHC had the necessary skills and experience to undertake the enhanced surveillance; however, the small team benefited from the support provided by SPC in running the surveillance. Additionally, the use of SAGES-OE was made easier through the partnership between SPC and JHU. Finally, there is a need to ensure effective connection with laboratory services to ensure that clinical sample collection more closely matches syndrome patterns.

### Conclusions and recommendations

The enhanced surveillance system used at the 8th Micronesian Games demonstrated: (1) the value of enhanced surveillance to provide public health security assurance during mass gatherings particularly in the face of significant, existing disease threats; (2) the benefit of web-based tools in improving the efficiency of the surveillance; (3) the potential for sustainable improvements to routine surveillance through leveraging the surveillance experience; (4) and improved health planning and informed decision-making that arise from the evidence base that is generated from the enhanced surveillance. Pohnpei was one of the first places in the Pacific region to implement the web-based SAGES-OE tool for mass gathering surveillance, and the enhanced surveillance implemented for the 8th Micronesian Games in Pohnpei provides a constructive model for future mass gathering surveillance across the Pacific and elsewhere.
